# Epigenetic alterations are associated with tumor mutation burden in non-small cell lung cancer

**DOI:** 10.1186/s40425-019-0660-7

**Published:** 2019-07-26

**Authors:** Liangliang Cai, Hua Bai, Jianchun Duan, Zhijie Wang, Shugeng Gao, Di Wang, Shuhang Wang, Jun Jiang, Jiefei Han, Yanhua Tian, Xue Zhang, Hao Ye, Minghui Li, Bingding Huang, Jie He, Jie Wang

**Affiliations:** 1 Department of Medical Oncology, National Cancer Center/National Clinical Research Center for Cancer/Cancer Hospital, Chinese Academy of Medical Sciences and Peking Union Medical College, Beijing, China; 2 Department of Oncology, Affiliated Hospital of Qinghai University, Xining, China; 3 Sinotech Genomics Ltd, Shanghai, China

**Keywords:** Genetics, Epigenetics, Non-small cell lung cancer, Tumor mutation burden, Checkpoint inhibitor therapy

## Abstract

**Background:**

To profile genomic and epigenomic of a naïve Chinese non-small cell lung cancer (NSCLC) cohort and investigate the association between tumor mutation burden (TMB) and DNA methylation (DNAm) to explore potential alternative/complimentary biomarkers for NSCLC immunotherapies.

**Methods:**

A total of 89 tumor tissues with matched normal tissues from Chinese NSCLC patients were collected and subjected to whole exome sequencing (WES). From comparison, each patient was evaluated for the TMB value and divided into high, medium and low TMB based on TMB tertile distribution and then relatively high and low TMB samples were selected and subjected to DNAm profiling.

**Results:**

Patients in the low (*n* = 30), medium (*n* = 29), and high (*n* = 30) TMB tertiles had 1.1–2.5, 2.5–4.1, and 4.2–13.9 mutations/Mb, respectively. A statistical directly association between differential methylation probes (DMPs) and TMB level was observed in our cohort (*r* = 0.63, *P* value =0.0003) and this was confirmed by using TCGA NSCLC dataset (*r* = 0.43, *P* value =0.006). Relatively high TMB group (*n* = 16, 7.5–13.9 mutations/Mb) harbors more differential DMPs while less in relatively low TMB group (*n* = 13, 1.1–2.4 mutations/Mb). Eight hundred fifty-eight differential methylation regions (DMRs) were found in relatively high TMB group. In addition, 437 genes show DNAm aberrance status in high TMB patient group and 99 have been reported as its association with lung cancer.

**Conclusion:**

To our knowledge, this is the first report for direct link between the methylome alterations and TMB in NSCLCs. High TMB NSCLCs had more DNAm aberrance and copy number variations (CNVs). In addition, the TMB distribution of Chinese NSCLCs population is lower than that of TCGA.

**Electronic supplementary material:**

The online version of this article (10.1186/s40425-019-0660-7) contains supplementary material, which is available to authorized users.

## Background

Lung cancer is the leading cause of cancer death worldwide and highly prevalent in China [1]. Approximately 85% of lung cancer cases are non-small cell lung cancer (NSCLC) [2]. Traditional target therapies have been effective against target population but they often suffer rapid relapse [3–5], such as target therapies against EGFR mutations [6], EMLA4-ALK fusion [7], and ROS-1 rearrangement positive [8]. Recent advances in immune checkpoint inhibitors (ICIs) [9, 10], including anti-PD-1 [11], anti-PD-L1 [12], and anti-CTLA4 [13] antibodies, may have the potential to transform cancer into chronical disease by relying on normalizing patients’ own immune system in tumor microenvironment. However, so far, not all lung cancer patients give an effective clinical response to ICI therapy even the positive PD-L1 expression in tumor tissue [14]. This demands an effective biomarker for ICI responding patient stratification.

Tumor mutation burden (TMB) has been proved to be effective in differentiating responding population of ICI therapies in multiple clinical studies. In addition, PD-L1 expression, microsatellite instability and deficient mutation mismatch repair have been used as companion diagnostic biomarkers for ICI therapy [15]. Tumor-infiltrating lymphocytes are another potential biomarker in tumor microenvironment [16, 17]. Among these biomarkers, TMB remains the most promising candidate up-to-date due to its relatively high positive screening rate.

Epigenetic modification, particularly DNA methylation (DNAm) has been linked to genomic instability, such as mutations in a DNA methyltransferase gene may cause chromosome instability in human and mouse [18, 19], and the LINE-1 hypomethylation has been found to associate with global loss of imprinting, which induce chromosomal instability in colorectal cancer and head and neck squamous cell carcinoma [20, 21].

However, direct correlation between DNAm status and TMB has not been addressed to date in NSCLC clinical samples. Here, we investigated DNAm profiles of a Chinese NSCLC cohort, together with whole exome sequencing (WES) data to explore their direct correlation with TMB. This can provide further insights for future novel biomarker developments for ICI therapies.

## Methods

### Patient cohorts

We have selected a total of 89 treatment naïve lung adenocarcinoma (LUAD) or lung squamous cell carcinoma (LUSC) patients from the Cancer Hospital, Chinese Academy of Medical Sciences & Peking Union Medical College who underwent definitive surgical resection before adjuvant therapy, including chemotherapy or radiotherapy. This study was approved by the Cancer Hospital, Chinese Academy of Medical Sciences & Peking Union Medical College, and performed in accordance with the Declaration of Helsinki Principles. All these samples were fresh frozen tissues which were under low temperature conditions (at − 80 °C). After obtaining informed consents, tumor tissues and their matched control were obtained for WES and DNAm profiling. To avoid the contamination of tumor tissue, all the matched normal tissues were collected at lobectomy edge. All samples had been subjected to pathology review for histological subtyping. The detailed clinical characteristics of these 89 Chinese Han population samples are summarized in Additional file 1: Table S1.

### WES and data processing

Sequencing protocol: DNA libraries for tumor and their matched control samples were prepared with standard protocol using MGIEasy Exome Capture V4 Probe Set capture kit (cat. No: 1000007745, https://en.mgitech.cn/article/detail/v4.html) with the capture region size 36 Mb. Pair-end sequencing (2 × 100 bp) was performed on BGI-Seq 500 instruments. Data processing: Alignment: The raw paired end reads were mapped to the human reference genome (hg19) using bwa-mem (version 0.7.16 with –M option: mark shorter split hits as secondary and the remaining setting was at default). Samtools v1.3.1 was used to sort and merge bam files from the same patient sequenced from different lanes. PCR duplicate read pairs were identified using biobambam (v.0.0.148). The quality control (all the sample QC files were available at https://drive.google.com/open?id=1HggApA8homvpF4xD2YOI3EQ2HsY3hS4S) was generated with FastQC (v0.11.8) and the post-alignment QC metrics information was shown in Additional file 1: Table S2. Variants calling: Variants calling was performed using a modified version of DKFZ-pipeline based on samtools mpileup and bcftools version 0.1.19 (pcawg-dkfz-workflow). Briefly, variants in the tumor sample were initially and used as query in the control sample. The raw calls were then annotated with various publicly available databases, including 1000 Genomes variants, ESP exon database, single-nucleotide polymorphism database (dbSNP), ExAC v.0.3.1 (non-TCGA variants), repeats and other elements. The functional consequence of the variants was predicted using Annovar [22] with UCSC Refseq annotations, followed by assessment of the variants in terms of their confidence and then classified into somatic or non-somatic calls. Only highly confident somatic variants with the following filtering criteria: Read Depth > =10, AF > =5%, Number of reads indicating mutation > = 3, were used for further analysis. TMB level is defined by two ways: one is as number of nonsynonymous coding somatic mutations (NOMs) per tumor, including single nucleotide variation (SNVs) and short insertion/deletion polymorphism (INDELs); the other is the number of mutations is proportion to the size of UCSC Refseq annotations (33.4 Mb). R/Biocondcutor package “maftools” [23] was used for visualization and summarization of MAF files from this study. TCGA WES somatic mutations**:** Confident somatic mutation calls derived from the WES data of the LUAD and LUSC cohorts were directly downloaded from the TCGA GDC Data Portal (https://portal.gdc.cancer.gov).

### Analysis of mutational signatures

Mutational signature analysis was conducted using the deconstructSigs package v1.8.0 [24]. All the detected somatic mutations including synonymous in the cohort were imported for signature analysis. In details, the frequency of 96 possible mutation types in trinucleotide context of each patient were first calculated in somatic mutation dataset. Normalization were then processed, according to the number of times each trinucleotide context is observed in our capture region. Finally, the weights of 30 known cancer mutation signature in COSMIC (https://cancer.sanger.ac.uk/cosmic/signatures) were generated by linear regression based on normalized frequency of each possible mutation types. Each weight indicates that how strongly can mutation signature influence the patient. Hierarchy cluster based on mutation signatures’ weights among patients were drawn by R package ‘pheatmap’ [25].

### Assessment of DNA methylation profiles

Five hundred nanogram of genomic DNA from each sample was bisulfite converted using the EZ DNA Methylation Kit (Zymo Research, Irvine, CA) and then analyzed on Infinium HumanMethylation 850 K EPIC BeadChip (Illumina, San Diego, CA) following the manufacturer’s instructions. The array features more than 850,000 methylation sites covering 96% CpG islands and 99% gene’s promoters. Raw data were analyzed using the “ChAMP” (Chip Analysis Methylation Pipeline for Illumina HumanMethylation450 and EPIC) package in R [26, 27] and all relevant parameters are default values. The differential methylated probe (DMP) of each sample was identified by the beta value of cancer and matched normal tissue with Benjamini-Hochberg (BH)-adjusted *P*-value < 0.05. R/Biocondcutor package “ConsensusClusterPlus” [28] was used for consensus clustering of Ilumina EPIC data. Bumphunter algorithms was applied to estimate regions for which a genomic profile deviates from its baseline value. Originally implemented to detect differentially methylated genomic regions between tumors and normal controls. By default, the progress of differential methylation region (DMR) finding was done on normalized beta value. The detected DMR and estimated *P* value (0.05 as cutoff value) was returned.

### Determination of copy number alterations (CNA) using the EPIC array and GO enrichment

The R/Bioconductor package ‘conumee’ [29] was employed to calculate CNAs based on the intensities generated using the EPIC array (using default settings). GISTIC [30] was then used to identified common deleted/amplified regions/genes (using default parameters). GISTIC is a tool that identifies genes targeted by somatic copy-number alterations (SCNAs) that trigger cancer growth. By classifying SCNA profiles as arm-level and focal alterations, this tool calculates the background rates of each category as well as delineates the boundaries of the SCNA regions. Aneuploidy score (AS) was calculated as is reported [31, 32], and the scores of each arm are − 1 if lost, + 1 if gained, 0 if non-aneuploid, and “NA” otherwise. For gene enrichment analysis, the function annotation tool from the DAVID website (https://david-d.ncifcrf.gov/) was used.

### Statistics

All statistical tests were conducted in R version 3.4.1 (The R Foundation for Statistical Computing, Austria). Unpaired t test was performed to evaluate the significance of TMB value between two groups (smoking: non-smoking, TP53+: TP53- and Chinese: TCGA LUAD/LUSC). Pearson’s correlation coefficient was calculated to evaluate the strength of correlation between DNA methylation and TMB levels. * stands for *P* value < 0.05. ** stands for *P* value < 0.01. *** stands for *P* value < 0.001.

## Results

### DNA methylation changes differently between high TMB and low TMB NSCLCs

**Fig. 1:**
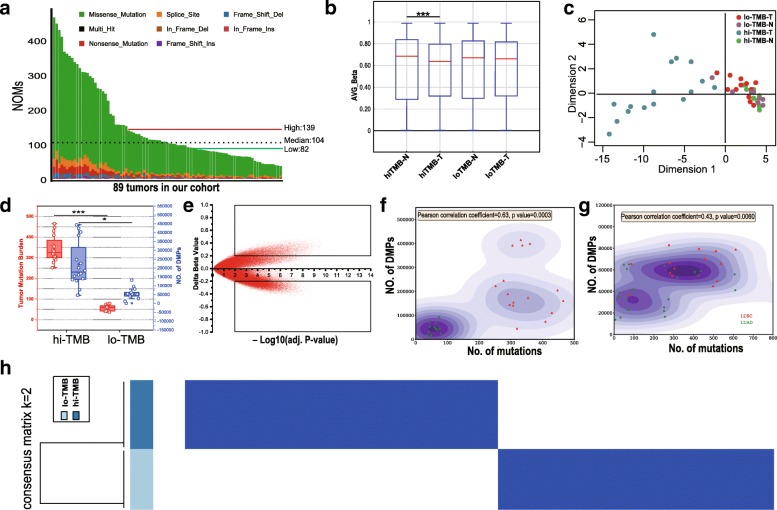
The methylome of relatively high TMB lung cancer is unique, and many DMRs are recurrent. **a** The NOMs for every patient (represented by the x-axis). Red/green lines indicate the high/low TMB cutoff in our cohort; Bar plot (**b**) and MDS analysis (**c**) of all CpG sites; **d** Identification of differences in DNAm between high TMB and low TMB groups. **e** Scatter plot between methylation changes (delta-beta value, high TMB tumors vs. controls) and corresponding -log10(BH adj. *P*-value) for total assessed 865,918 sites was shown. CpG sites with deltabeta < 0.2 and -log10(BH adj. *P*-value) < 2 were defined as MVPs. The upper square indicates hypermethylated MVPs, and the bottom square indicates hypomethylated MVPs compared with controls; **f** The comparation of differently methylation sites and TMB of NSCLCs (LUAD/LUSC) in our cohort; **g** The comparation of differently methylation sites and TMB of NSCLCs (LUAD/LUSC) in TCGA dataset; **h** Consensus clustering of the DNA methylation reveals high and low TMB lung cancer groups of DNA methylation. 293 informative probes with strict screening parameters (s.d. > 0.2 between high and low TMB group, s.d. < 0.1 in high or low TMB group, |delta beta| > 0.2, BH adjusted *P* value < 0.05) were used for the consensus clustering

These 89 patients included in this study were consist of 65 LUAD as well as 24 LUSC patients. From WES data analysis, only high confidence nonsynonymous somatic mutations (Tumor DP > =10, AF > =5%, NO. of reads indicating mutation > = 3) were processed for TMB assessment. The mean coverage is achieved at 167×, 161× in tumor samples and normal samples, respectively. More than 90% of targeted regions with coverage > 10× were found in 87/89 pair samples. TMB distribution showed a median number of 104 NOMs per tumor, ranging from 37 to 465 (Fig. 1a). Consistent with the approach of the clinical trial CheckMate 026 [33], we classified our cohort by high (139–465), medium (83–136) and low (37–82) NOMs or low (1.1–2.5), medium (2.5–4.1), and high (4.2–13.9) mutations/Mb. In order to further explore the relationship between DNAm and TMB, 13 relatively low (37–79 mutations or 1.1–2.4 mutations/Mb) and 16 relatively high (252–465 mutations or 7.5–13.9 mutations/Mb) TMB samples were selected for subsequent methylation level detection. Due to the insufficient amount of DNA after the WES experiment, these samples were not selected successively. Unless specifically mentioned, the high or low TMB group in the following text represents the relatively high TMB group and the relatively low TMB group.

DNAm profiles in tumor samples and its matched normal controls were measured using the Illumina Infinium HumanMethylation EPIC BeadChip platform (850 K), which evaluates methylation status of 865,918 CpG sites covering key features of the human whole genome. The R package ‘ChAMP’ for Illumina EPIC was applied for data analysis. The beta-value was chosen as a measure of the methylation level, which ranges from 0 (no methylation) to 1 (complete methylation). Based on the methylation level of 865,918 sites, differential global methylation status (unpaired t test, *P* value < 0.001) was seen between high TMB group (median beta-value of 0.643) and its matched controls (median beta-value of 0.629), while 0.631 and 0.629 in low TMB group (Fig. 1b**)**. The methylation status in tumor tissue comparing normal tissue was different between high and low TMB groups, and this was further confirmed by multidimensional scaling (MDS) analysis of the CpGs (Fig. 1c). Data of tumor tissues cluster separately from the normal tissues in high TMB patients, thereby indicating a different global methylation pattern. However, in low TMB patients, the tumor tissues cluster overlaps with their corresponding normal tissues, which indicates stable epigenomic profile between tumor and normal tissues in low TMB patients. Cluster analysis also revealed variable global methylation patterns in high TMB group comparing to low TMB group. 292121 significant DMPs with a BH-adjusted *P*-value below 0.05 were found while none in low TMB group. Box plot analysis further shows that high TMB group (median TMB =343) harbors significantly more differential methylation locis (31,279~391,387, with median of 188,637) with |delta beta| > 0.2 than low TMB group (median TMB =62; 10,479~92,932, with median of 43,340) in Fig. 1d. We obtained differentially methylated region (DMR) in high TMB group in a total number of 858 regions (Additional file 1: Table S3), while none in low TMB group. To exclude that the observed differences in DMPs between high and low TMB samples are driven by different leukocyte enrichment, R/Bioconductor package “minfi” [34] was applied for cell type composition. The results (Additional file 2: Figure S1) showed that no significant differences were observed in CD8T, CD4T, NK, Bcell, Mono and Gran cells between high and low TMB samples.

Methylation differences between high TMB tumor and matched normal tissues were calculated as delta-beta and plotted against corresponding −log10 (BH-adjusted *P* value), as shown in Fig. 1e. With a consideration of so much DMPs and further analysis in high TMB group, we defined CpG sites with |delta-beta| > 0.2 and BH-adjusted *P* value < 0.01 as methylation variable positions (MVPs). From the over 850,000 informative probes, 61,633 MVPs were identified, representing < 7% of the total sites surveyed and top 3000 MVPs were shown in Additional file 1: Table S4. There were more hypomethylated CpG sites (44,718 MVPs, delta-beta <− 0.2, bottom square) than hypermethylated CpG sites (16,915 MVPs, delta-beta > 0.2, upper square).

After comparing each cancer and matched normal tissues of differential methylation data in high or low TMB groups, we found that high TMB patient samples contain more DMPs (Pearson correlation coefficient = 0.63, *P* value =0.0003) comparing to low TMB patient samples (Fig. 1f). For further validation, we execute of analysis of tumor mutation data and DNA methylation data of 39 TCGA NSCLCs with high- (top 20% by TMB) and low- TMB (bottom 20%) as is shown in Fig. 1g. Positive correlation (Pearson correlation coefficient = 0.43, *P* value =0.006) was also found between NOMs and DMPs in such independent dataset. By analyzing LUAD or LUSC samples separately, the DMPs of LUAD or LUSC was significantly correlated with TMB as is shown in Additional file 2: Figure S2. The same analysis in the TCGA NSCLCs database is consistent with this result (Top 12 high TMB vs bottom 12 low TMB: *P* value =0.0026, Mann–Whitney test) as is shown in Additional file 2: Figure S3. To further identify methylation sites that distinguish high TMB and low TMB lung cancer, we utilized k-means consensus to perform cluster to these 29 primary lung cancer tissue samples from our cohort with 293 most variable methylation loci (s.d. > 0.2 between high and low TMB group, s.d. < 0.1 in high or low TMB group, |delta beta| > 0.2, BH adjusted *P* value < 0.05, Additional file 1: Table S5). We observed two distinct groups of samples (Fig. 1h), which were correlated to the high or low TMB lung cancer groups.

### High TMB NSCLC patients harbor more structural variation of CNV

**Fig. 2:**
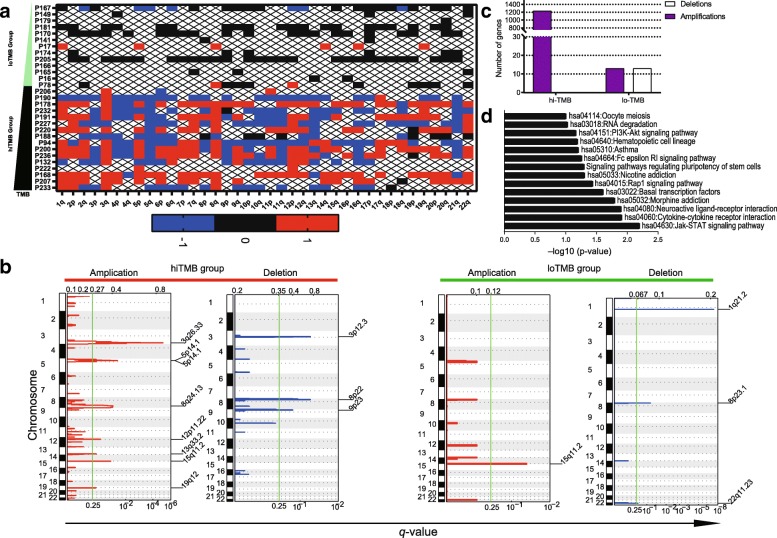
Numerous copy number amplifications characterize high TMB cancers. **a** CNA profiles of high TMB and low TMB group: heatmaps of aneuploidy score calculated via the intensities of the EPIC array (each tumor versus average normal). The scores of each arm are − 1 if lost, + 1 if gained, 0 if non-aneuploid, and “NA” otherwise; **b** Amplifications: q values of amplifications of all tumors of high−/low- TMB lung cancer tumors. Deletions: q values of deletions of all tumors of high−/low- TMB lung cancer tumors; **c** Confirmed number of genes that map to significantly amplified or deleted regions; **d** GO enrichment analysis showing the enriched pathways of amplified and deleted genes in high TMB lung cancers

CpG hypomethylation status has been reported to be related to genetic instabilities, and global hypomethylation in tumor indicates more genomic instabilities [35]. We checked the copy number variation (CNV) in high TMB and low TMB group using aneuploidy score (AS) and found high TMB lung cancers possess more structural variation of CNV, while low TMB ones appeared to retain more stable genomic structural profile (Fig. 2a**,** Additional file 1: Table S6). The analysis results of TCGA database are consistent with our cohort study (*r* = 0.18, *P* value =1 × 10^− 8^, Pearson correlation analysis) as is shown in Additional file 1: Table S7 and Additional file 2: Figure S4. Compared to the low TMB group, the high TMB NSCLC group exhibited more genomic deletions and amplifications (Fig. 2b), particularly a gain in chromosome arm 3q (especially 3q26) and a loss of chromosome 3p (especially 3p12). Frequent localized amplifications within chromosomal regions 8q24, 12p11, and 15q11 loci and deletions within 8p22 and 9p23 were also detected. A total of 1237 genes (Additional file 1: Table S8) were significantly mapped to these amplified regions, whereas no genes could be significantly mapped to the deleted regions (whole chromosomal arm deletions were excluded from the analysis) (Fig. 2c). Several of these recurrent CNAs exhibited high chromosomal instability, possibly lead to TMB value increasing. The 1237 genes in CNA regions were also assessed in terms of Gene Ontology enrichment by DAVID, which revealed that the pathways of Jak-STAT signaling (hsa04630) and cytokine-cytokine receptor interaction (hsa04060) were highly represented in Fig. 2d.

### 437 Genes’ promoter regions showed DNAm aberrance status in high TMB NSCLCs

**Fig. 3:**
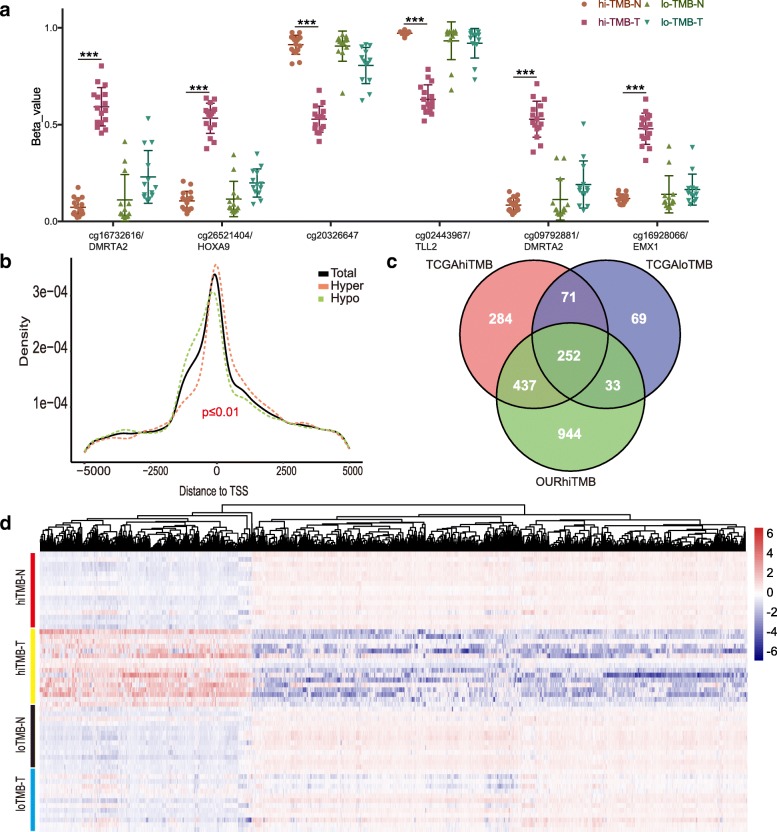
The relationship of HOX gene methylation status and TMB. **a** Top 6 differential methylation sites in hi-TMB comparing with low TMB group. cg16732616/DMRTA2, cg26521404/HOXA9, cg20326647/intergenic region, cg02443967/TLL2, cg09792881/DMRTA2 and cg16928066/EMX1 were significantly methylated in high TMB tumor tissues; **b** In hi-TMB lung cancer, CpGs that are hypomethylated are more likely to be found immediately upstream of the TSS and within the 1st exon, CpGs are hypermethylated (*P* value < 0.01, two-tailed Wilcoxon rank-sum test); **c** Venn diagram analysis revealed 437 genes associated with high TMB; **d** The heatmap of the all methylation probes related to 437 genes in high TMB, low TMB NSCLCs samples and the matched normal controls

In high TMB group, more significant DMPs was found and Fig. 3a shows the top 6 DMPs (cg16732616/DMRTA2, cg26521404/HOXA9, cg20326647/intergenic region, cg02443967/TLL2, cg09792881/DMRTA2 and cg16928066/EMX1) as representives. We also explored the distribution of the DMPs and found hypermethylated DMPs were located closer to transcription start site (TSS), whereas the hypomethylated DMPs was shifted slightly to upstream of TSS (Fig. 3b). We focused on the MVPs with No. > 3 at the promoter region referring to TSS1,500, TSS200, 5′-UTR, and 1stExon to discover significant differential methylated gene and found 1666 genes, in which HOX family genes (26 out of 39 [36, 37]) were most effected (Additional file 2: Figure S5). In order to further exclude inappropriate genes caused by the number of samples, the same analysis was carried out in the TCGA NSCLC database, and Venn analysis (Fig. 3c, Additional file 1: Table S9) showed that there were 437 genes associated with the state of high TMB. The heatmap plot (Fig. 3d) analyzed all the 8703 probes from the 850 k chip related to these 437 genes, and the results show that they are significantly different in the high TMB group. The same analysis was performed on 4916 probes from 450 k chips in the TCGA database, and the results (Additional file 2: Figure S6) were consistent with our cohort study. To further analyze the relationship between these 437 genes and lung cancer, we used DisGeNET [38], a database of gene-disease associations, to analyze the network of these genes, and found that there were 99 genes, related to “Neoplastic Process” of lung (Additional file 1: Table S10).

### Chinese NSCLCs have lower NOMs than TCGA LUAD/LUSC

**Fig. 4:**
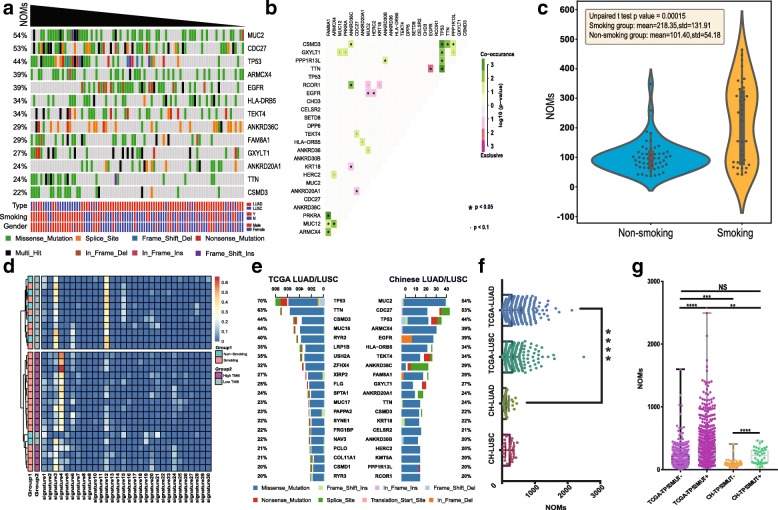
Comparative analysis between Chinese and TCGA lung cancer populations. **a** The top 13 genes with most frequent mutations in our cohort with the decrease in NOMs; **b** Somatic interactions in our cohort. Such mutually exclusive or co-occurring set of genes can be detected using the somaticInteractions function in R/Bioconductor package ‘maftools’, which performs pair-wise fisher’s exact test to detect such significant pair of genes; **c** Comparison of TMB levels between smoking and non-smoking group. Unpaired t test *P* value =0.00015, Smoking group: mean = 218, Non-smoking group: mean = 101.40; **d** Heatmap plot to interpret the possible associations of mutation signature and TMB classification. Generally, high TMB patients with smoking history display a strong signal on signature 4 (the known signature associated with cigarette). Another high TMB group without smoking history display a dominant weight on signature 3 (The signature probably caused by failure of DNA double-strand break-repair in homologous recombination). Signature 3 and signature 12 occurred simultaneously in low TMB patients, regardless of smoking status; **e** The differential patterns of mutation between Chinese lung cancer population and TCGA LUAD/LUSC; **f** The comparation of Chinese LUAD/LUSC and TCGA LUSC/LUAD NOMs; **g** The relationship of TP53 and NOMs in Chinese NSCLSs and TCGA NSCLCs

The TMB distribution of Chinese NSCLCs has not been well reported in the literature, therefore its description may provide insights for pharmaceutical companies or diagnostic industry to adjust their commercial strategy in China. Recent studies had demonstrated that loss of TP53 function increased genomic instability [39, 40]. We further investigate the mechanism of these differences between these two populations based on genetic alterations. An important driver gene of Chinese NSCLCs, EGFR mutations, which are closely related to the efficacy of molecularly targeted therapy (EGFR TKIs), have been reported to negatively correlate with TMB value [41, 42]. Heatmap plot shows that frequently mutated genes such as TP53 gene, which tends to been enriched in high TMB group (top 30 samples, range: 139–465 NOMs) in lung cancer; EGFR mutants in low TMB (bottom 30 samples, range: 37–82 NOMs), and patients with co-existence of TP53 and EGFR mutations in the intermediate TMB level (median 29 samples, range: 83–136 NOMs) (Fig. 4a**)**. Many disease-causing genes in cancer are co-occurring or show strong exclusiveness in their mutation pattern with high TMB. In our study, gene set TP53, CSMD3, GXYLT1, PPP1R13L and TTN shows a strong co-occurrence and gene set EGFR, TTN, MUC2 and HERC2 shows a strong exclusiveness in high TMB group (Fig. 4b). It was been confirmed in our study that the high TMB samples was mostly LUSC with smoking habit. Our study confirm that smoking was also a key factor associated with TMB **(**Fig. 4c**)**. We evaluated 30 known mutational signatures for different carcinogens in COSMIC database, including UV light or tobacco by calculating the frequency of specific mutation types in trinucleotide [43]. Consistent with previous findings, we observed that high TMB patients displayed distinct mutation signatures comparing to low TMB patients as is shown in Fig. 4d. Signature 4 was the dominant mutation pattern in high TMB patients with smoking history, while high TMB patients without smoking history contained relatively stronger mutation pattern in Signature 3. Signature 3 and signature 12 occurred simultaneously in low TMB patients, regardless of smoking status. Since signature 4 is a well-known tobacco-related signature characterized by transcriptional strand bias in C > A mutations, it matches the phenotype of smoking-history among high TMB patients. The failure of DNA double-strand break-repair in homologous recombination indicated by signature 3 may confer high mutation ability to patients without smoking history. Regarding to low TMB patients, efforts are needed to investigate the etiology of the strong signal in signature 12 with T > C substitutions.

Interestingly, the frequency of TP53 and EGFR mutations between our cohort and TCGA cohort was different (TP53: Chinese 46%, TCGA 70%, EGFR; Chinese 39%, TCGA 17%) (Fig. 4e). This result was consistent with previous study in Chinese lung cancer population [44]. TMB distribution (median value =104 NOMs per tumor) in our cohort is lower than TCGA LUAD/LUSC (median value =176 NOMs per tumor). The TMB value of LUSC was significantly (unpaired t test, *P* value < 0.001) greater than the value of LUAD **(**Fig. 4f). After further analysis, it indicates that TP53 mutations significantly affect TMB level in both Chinese NSCLCs between TP53+ and TP53- mutation group (unpaired t test, *P* value < 0.001) and TCGA LUAD/LUSC (unpaired t test, *P* value < 0.001) (Fig. 4g). In the analysis based on Asian, Black, White populations from TCGA database, the results (Additional file 2: Figure S7 and S8) were consistent with our findings: Asian (*n* = 17, TP53: 65%, EGFR: 24%, mean NOMs: 151), Black (*n* = 81, TP53: 70%, mean NOMs: 292) and White (*n* = 731, TP53: 62%, mean NOMs: 251).

## Discussion

Although linkage between methylation change and chromosomal instability has been widely reported, a direct link between differential methylation and TMB values has not been directly measured in NSCLC population. The results of the NCT02259621 trial indicated that TMB may be used as a biomarker for the pathological responses to the PD-1 blockade. Around 2 to 4 weeks after neoadjuvant nivolumab treatment, a rapid expansion of mutation-related, neoantigen-specific T-cell clones derived from a primary tumor that showed a complete response on pathological assessment, was observed in the peripheral blood of 8 out of 9 patients and a number of these clones were not identified prior to the nivolumab administration. Since then, TMB has been well reported to serve as a biomarker for stratifying patients for PD-1/PD-L1 therapies. Interestingly, a recent study suggested that methylation pattern change may also serve as a prognosis biomarker for anti-PD-1 treatment [45]. Therefore, we conducted this study to investigate the correlation between TMB and DNAm profile. Our study revealed the significant correlation of DNAm and TMB in NSCLCs. To our knowledge, this is the first NSCLC cohort study to directly link the methylome alteration to TMB.

Methylome-wide analysis revealed widespread changes in lung cancer-associated DNAm patterns, particularly in high TMB cancer tissues. Earlier studies showed that DNA hypomethylation within the coding regions of genes is often associated with genome instability and higher mutation rate. However, those investigations were mostly performed in vitro in cell lines [46]. Our study results confirmed these findings with a comparison of primary NSCLC to matched normal tissues in our cohort and TCGA dataset. Based on these results, differential methylated target regions may work as a potential biomarker along with TMB or even as an alternative approach since accurate measurement of TMB demands a relatively large panel to harbor significant genomic variations which is quite expensive while methylation profiling methodology are more robust and reasonably economic. More comprehensive study on methylation regions with large scale PD-1/PD-L1 therapeutic patient samples with clinical outcome need to be conducted to lock down a panel of genes that methylation status correlate with ICI benefit.

To investigate the correlation between DNAm and TMB more extensively, we investigated 1666 genes that are significant differential methylated in our cohort. Among these genes, an important cluster of genes with hypermethylated CpGs is HOX gene family and its hypermethylation status has been reported to be associated with the low expression of HOX in lung cancer [47]. Unfortunately, such significant differences in the HOX gene family were not observed in TCGA database, so that further studies are needed to explore the function of the HOX family genes. We need to take this conclusion very cautious since the dataset is relatively small, and we only analyzed relatively high and low TMB NSCLC samples with the intermediate TMB samples to be excluded (due to samples shortage). However, our data raised a hypothesis that maybe a gene family methylation status or maybe a methylation panel can be served as a potential biomarker for ICI therapy. By integrating with the TCGA database, our study also revealed 437 potentially differentially methylated genes associated with high TMB, including 99 genes that are closely related to lung cancer disease. Sine the cost for methylation panel assessment are much lower than TMB assessment, some genes’ methylation status may be a potentially promising biomarker. Nevertheless, further studies with larger size, more importantly with PD-L1 clinical outcome, are needed to further select and confirm biomarkers to improve precision management of NSCLCs ICI therapies.

EPIC 850 K arrays were employed for copy number analysis in parallel to DNAm analysis with the same DNA specimen. The 850 K array probes are as robust and sensitive as SNP arrays, resulting in CNA calls for its more wider probes coverage (> 850,000 CpGs). High TMB NSCLCs exhibited an imbalanced genome with several chromosomal gains and losses, whereas low TMB NSCLC samples showed much lower level of chromosome instability. We also confirmed that high TMB LUSC samples harbor numerous CNAs as well as aberrantly methylated sites and exhibit distinct mutational signatures.

When it was mentioned, currently, it was difficult to define an exact TMB value for its real role on ICI therapy effect prediction, although it has been explored so much. There are extensive investigations on TMB distribution on Caucasian NSCLCs, not much data has been shown on Chines NSCLCs. Our data indicated that the TMB distribution of the Chinese NSCLCs population was significantly lower than the TMB range observed from TCGA LUAD/LUSC database. One plausible reason is that Asian, Black and Caucasian races tend to display different frequencies and patterns of tumor mutations. For example, Chinese lung cancer patients tends to harbor a much higher frequency of EGFR mutations. It has also been observed that TMB is much lower in EGFR mutated patients both in our cohort and TCGA dataset and the presence of driver alterations may provide clinically useful predictors of response to anti-PD-1/anti-PD-L1 therapies [48].

## Conclusions

In our study, our results show that Chinese NSCLCs population has lower TMB level than TCGA LUAD/LUSC because of the higher mutation rate of EGFR but lower in TP53. However, the necessity to adjust the recommended TMB threshold for personalized lung cancer immunotherapy remains unclear and only clinical results can give a definitive answer. Another complication is that all patients in our study were diagnosed with NSCLC at an early stage and thus have not received any systemic treatment, including chemotherapy, targeted therapy, or ICI therapy, this may also cause different TMB distribution. Our data also confirmed the association between TP53 mutations and high TMB levels in the Chinese and TCGA LUAD/LUSC, and the association between cigarette smoking and high TMB levels. Nevertheless, our study will draw more attention on TMB cutoff adjustment on PD-1/PD-L1 therapy on Chinese NSCLCs.

## Additional files

Additional file 1 : **Table S1.** Clinical characteristics of 89 NSCLCs in this study. **Table S2.** The post-alignment QC metrics information of WES. The post-alignment QC metrics information of WES including expected depth, the mean target coverage tumor and matched normal controls and the percentage of duplicates read, off targets and not mapped reads, GC content were provided. **Table S3.** 858 DMR in high TMB group comparing to matched normal controls. **Table S4.B**A list of the top 3000 MVPs. **Table S5.** 293 methylation sites that can distinguish between high or low TMB. **Table S6.** ASs and NOMs for each sample of our cohort. **Table S7.** ASs and NOMs for each sample of TCGA. **Table S8.** The 1237 gene list for the GO analysis with location info. **Table S9.** The gene list with the No. of MVP > 3 in our cohort and TCGA database for the Venn plot analysis. **Table S10.** Ninety-nine genes involved in “Neoplastic Process” of lung within DisGeNET database from 437 gene list. (RAR 1336 kb)

Additional file 2 : **Figure S1.** Cell type composition by sample type. **Figure S2.** The comparation of DMPs in high TMB or low TMB based on lung cancer type LUAD or LUSC in our cohort. **Figure S3.** The comparation of DMPs in high TMB or low TMB in TCGA NSCLCs. **Figure S4.** The correlation of ASs with NOMs in TCGA database**. Figure S5.** 4 HOX family gene cluster’s methylation status in our cohort. **Figure S6.** Heatmap plot of all the 437 genes’ methylation probes in TCGA database. **Figure S7.** Subgroup analysis of NOMs based on ethnicity (ASIAN, BLACK and WHITE) in TCGA database. **Figure S8.** The first 20 high frequency mutated genes of ASIAN, BLACK or WHITE population in TCGA database. (RAR 16686 kb)

## Data Availability

The datasets generated and/or analyzed during the current study are available from the corresponding author on reasonable request.

## Abbreviations

BH: Benjamini-Hochberg
CD4T: Cluster of differentiation 4 positive T cell
CD8T: Cluster of differentiation 8 positive T cell
CNV: Copy number variation
CTLA4: Cytotoxic T-lymphocyte–associated antigen 4
DMP: Differential methylation probe
DMR: Differential methylation region
DNAm: DNA methylation
EGFR: Epidermal growth factor receptor
FDR: False discovery rate
GO: Gene ontology
HOX: Homoeobox
INDEL: Insertion/deletion polymorphism
Jak-STAT: Janus kinase/signal transducers and activators of transcription
LUAD: Lung Adenocarcinoma
LUSC: Lung Squamous Cell Carcinoma
MVP: Methylation variable position
NK: Natural killer cell
NOM: Number of mutation
NSCLC: None small cell lung cancer;
PCR: Polymerase chain reaction
PD-1: Programmed cell death protein 1
PD-L1: Programmed death-ligand 1
SCNA: somatic copy-number alteration
SNV: Single nucleotide variation
TMB: tumor mutation burden
TP53: Tumor protein p53
TSS: Transcription start site
WES: Whole exome sequencing
